# A Contribution to the Study of the Therapeutical Use of Ipecac

**Published:** 1874-09-15

**Authors:** H. Chouppe


### Translations.

### A CONTRIBUTION TO THE STUDY OF THE THERAPEUTICAL USE OF IPECAC.

By Dr. H. Choupe.

*Translated from Le Progres Medical, by J. H. Etheridge, M. D.*

PHTHISICAL SWEATS.—After having seen ipecac yield good results in most of the cases of diarrhœa where I had used it, I determined upon assuring myself if it would not act the same in the sweats of tuberculous patients, in diminishing the quantity of the secretion. In the first part of this article I have given the cases separately, and subsequently appended reflections upon their observations;. adhering to this method, often tedious to a lecturer, I will divide my observations into groups, and each of these will be followed by short reflections :

GROUP I.

*Cases in which the results were wholly favorable.*

Case 1. Ward Saint Basile, bed seven. Female, aged thirty-seven. Admitted to confinement July, 1873. She began to cough during pregnancy, and had many hæmoptyses. Immediately after her confinement she had an intense bronchitis, which necessitated very energetic treatment. After this time the patient had numerous attacks of bronchitis, but in the interval well-marked signs of pulmonary phthisis, in the first stage, were detected in the top of the left lung. From this time profuse sweats, very abundant at night, supervened, compelling her to change her linen and fatiguing her much. This patient had never had diarrhœa.

Nov. 2. Ordered two injections of ipecac; up to this time the tannate of quinine in 4 gramme doses, and tannin itself in 40 centig. doses had been successively used, with no success in controlling the sweats.

Nov. 3. The sweats were diminished at least one-half. This morning only one injection ordered.

Nov. 4. The patient sweat none last night. Same treatment. The same condition continued the 5th and 6th. On the 7th the injection was stopped.

Nov. 8. The patient sweat a little in the night; half injection ordered.

Nov. 9–10. Same condition; a little sweating; continue treatment.

Nov. 11–12. Almost no sweating.

Nov. 13. No sweating; clysters discontinued.

Nov. 14. No sweating. Menses reappeared. General state better.

Nov. 15. Finds herself better. Says digestion is easier. The sweats had not reappeared up to Dec. 31, 1873. During this period the phthisis had not made any progress.

Case 2. Ward St. Louis, bed 13.

Male, aged thirty-seven years; admitted Nov. 29, 1873. Pulmonary phthisis, first stage; six months' duration. Night sweats of the upper part of the body for the past three months, recurring every night, with fever, about midnight, lasting two hours; patient obliged to change linen.

Dec. 4. Ordered a potion of ipecac decoction, in three parts; about eight o'clock nauseated; less sweat in the night.

Dec. 5. Same treatment; same effect.

Dec. 6. No sweating; patient vomited once.

Dec. 7. No sweating; ipecac stopped.

Dec. 8–9 and 10. Patient has not sweat; has gone to Vincennes, feeling much better.

Case 3. G., aged 41 years; admitted Nov. 19, 1873. Ward St. Louis, bed 10. This patient had, as a first symptom, a pleurisy, in July last. For past three months he has had such night sweats that he does not rest; is obliged to change linen on account of them. This phenomenon occurred regularly every night. The sweats are preceded by a sensation of heat, rather active. The physical signs are those of the first stage (prolonged expiration, dullness on percussion, etc.) at the top of the right lung. In the night of the 19th and 20th Nov., the patient experienced sweats, with the same characteristics as formerly.

Nov. 20. One clyster of ipecac at night.

Nov. 21. Sweat much less; had no fever in the night; continue treatment.

Nov. 22–23. The patient has not sweat; clyster of ipecac not given on night of 23rd inst.; no sweating on 23rd and 24th.

Nov. 25. The patient had a little sweat in the night; ordered a new clyster of ipecac. The injections of ipecac were continued every night regularly, till Dec. 2nd. Since Nov. 26th the sweats have not returned. On the 1st of Dec. the patient began to suffer from a copious diarrhœa, with rectal tenesmus. Clysters discontinued, and on the 3rd of Dec. the diarrhœa was cured spontaneously.

Up to Dec. 17th, no sweats returned. At this date a veritable diarrhœa came on, which had not the rectal characteristic which accompanied the former attack, and it was speedily cured by three injections of ipecac; up to Dec. 31st, 1873, no sweating had occurred.

(Obs.)

In the three preceding cases, we had to do with phthisical patients, in the commencement of their disease; notwithstanding we all know that even in this stage sweats are often very obstinate, yet we see that in three patients they were easily cured. I wish still further to call attention to the fact that, under the influence of this indication, we have seen, in cases Nos. 1 and 2, the fever which preceded the sweats rapidly diminishing, which produced a notable amelioration in the general condition of the patients.

Case No. 4. Ward St. Basile, bed 28. Admitted Sept. 15th, 1873. Pulmonary phthisis, third stage, chronic form; night sweats very abundant for many months. Every night patient obliged to change linen.

Nov. 9. Half clyster of ipecac.

Nov. 10. A little less sweating; patient not obliged to change linen in the night; same treatment.

Nov. 20. No sweats; injections of ipecac stopped. Sweats had not reappeared up to 31st of Dec. Before using ipecac, the salts of lead and tannin had been used in vain.

Case 5. Ward St. Louis, bed 3. Admitted Oct. 23d, 1873. Pulmonary phthisis in third stage; large cavities at top of both lungs. This patient was not subject to diarrhœa, but for six months, without missing a single night, he had soaked the pillows, so that his wife had to occupy another bed. The sweats recurred regularly about midnight, and were preceded by a light flush of fever. In the nights of the 23rd and 24th, he had sweats as abundant as formerly. Oct. 24th, ordered an injection of ipecac.

Oct. 25. The patient did not sweat during the night: ordered two injections of ipecac.

Oct. 26. No sweats; discontinued the clysters.

Oct. 29. Sweats not reappeared; general condition better, and at his own request the patient left town for Vincennes.

Case 6. Ward St. Louis, bed 19. Admitted Nov. 26th, 1873. Pulmonary phthisis, third stage. Began eight months ago. Sweat considerable and daily for three months.

Nov. 27. Ordered two clysters of ipecac.

Nov. 28. Patient has sweat less.

Nov. 29. Almost no sweating.

Nov. 30 and Dec. 1. No sweating; stopped injections.

Sweats had not reappeared up to the 16th of December, at which time the patient left the hospital in a much bettered condition.

(Obs.)

Here the results were always complete and decisive. We see, in running over this epitome, that the sweats have not only *not* reappeared, so long as the injections of ipecac were continued, but, what is still more important, that an amelioration persisted, in two cases, more than a month; in the other four, a considerable length of time after the cessation of the treatment. It is almost never the case that one obtains, even with the best means, results so favorable.

We furthermore see, that in cases Nos. 1 and 4, other treatments had been used in vain, before resorting to ipecac. It would seem that in some cases at least, this means may be more energetic than the medicines that were used.

One objection which may be offered, to which I have responded by the foregoing facts, is the following: We frequently see patients, especially consumptives, who, upon their admission to the hospital, without receiving any treatment, have experienced, simply from the change in conditions, a perceptible improvement, and have seen many symptoms disappear. Cases Nos. 2, 3, 5 and 6, can, strictly speaking, come within the scope of this critique, and though one may argue from the duration of the symptom before the patient's admission to the hospital, yet the objection can here be said to be possessed of all its value.

But cases Nos. 1 and 4 answer here convincingly; in these cases the patients really remained in the hospital many months without any changes in their hygienic condition, and in these especially was the result quite satisfactory and decisive, if it were not so in the other four cases. I could have waited, in all these cases, a few days, before beginning treatment, for the patient to become acclimated; I acknowledge that the idea did not occur to me, and, that in view of the intensity of the sweatings and of the uncomfortableness they produced, I concluded to act promptly.

It might, furthermore, be thought that the injections of ipecac act in sweatings only by means of the tannin which they hold in solution. To this argument I propose to respond later in this article, both by a chemical analysis of the injections and by direct experiment. I am glad to simply state, in passing, that the ipecac clysters have sufficed to arrest the sweats in two cases where tannin, given in rather large doses, produced no amelioration whatever.

### Group II.

*Cases in which the results have been favorable, but less prolonged.*

Case 7. Ward St. Louis, bed 24. Admitted Dec. 17th, 1873; aged thirty years. Pulmonary phthisis, third period. Cavity in left lung and crepitus at the top of the right lung. Internal otitis on the left side. The disease seems to have sprung up within three months. For three months the night sweats have been very abundant; patient obliged to change linen every night; fever in the evening.

Dec. 18. One clyster of ipecac.

Dec. 19, 20 and 21. Patient has not sweat.

Dec. 22. Sweats reappeared, though less abundant; injections were not renewed.

Dec. 31. Sweats are again copious.

Case 8. M., aged 47 years; admitted Oct. 30th, 1873; Ward St. Louis, bed 28. Pulmonary phthisis seems to have sprung up within the past five months. Within this period the patient has had hæmoptyses very abundant and repeated. The disease has now reached its second period, and crepitus, easily detected, at the top of the left lung, exists.

One of the symptoms which fatigues the patient the most, is the abundant night sweats, recurring especially the second half of the night, and which compel the patient to change his linen many times.

Nov. 16. Sweats not modified since the patient came into the hospital. Gave him one injection of ipecac to-day.

Nov. 17. Sweats considerably less; continue.

Nov. 18. Amelioration continues.

Nov. 19. The patient still sweats a little, but the sweatings appear only after a calm refreshing sleep, which lasts during the major part of the night. He insists upon this fact especially: that since he began taking the clysters at night he has less fever; less heat at night; that his sleep is more calm; that he sweats but little, especially about the head, and that he is not obliged to change his linen as formerly.

Nov. 21st and 22nd. Treatment continued and results are the same.

Case 9. A young woman, aged 22 years. Admitted Nov. 19th, 1872; Ward St. Basile, bed 4. Chlorotic for the past two years; she has coughed for three months only; cough dry, with some physical signs of tubercle of the right lung; since the advent of her illness she has been subject to very abundant night sweats, which fatigue her much.

Nov. 20. Injection of ipecac.

Nov. 21. Injection produced no effect.

Nov. 22. Patient presented all the signs of an acute gastric attack, (embarras.) Ipecac withheld.

Dec. 4. Symptoms of gastric difficulty have nearly disappeared; appetite has returned a little, but the sweats at night persist with the same characteristics. Ordered a potion of ipecac at 8 P. M.

Dec. 5. Patient vomited at three different times, but sweat less during the night; she was not obliged to change her linen as she has done heretofore, every night.

Dec. 6. Vomited once only; almost no sweating.

Dec. 7 and 8. Vomiting all the time; almost no sweating. Potion replaced by the injection at night.

Dec. 10 and 11. Same condition.

Dec. 12. Return to clysters again.

Dec. 20. The patient gives, verbatim, this account of herself: "The sweats are scarcely appreciable."

Dec. 22. She left for Vesinet to-day.

Case 10. Patient, female; aged 22 years. Admitted Dec. 13th, 1873; Ward St. Basile, bed 13. Pulmonary phthisis, first stage. The patient has had night sweats for three months.

Dec. 15. Ipecac clyster ordered at night.

Dec. 16. She has not sweat.

Dec. 17. Sweats have not returned. Ipecac suppressed.

Dec. 18. No sweats.

Dec. 19. Reappeared a little; clysters of ipecac were not given again, as the patient did not endure them very well.

Dec. 25 to 30. The sweats which, during the interval that has just elapsed, have resisted the action of tannin, have been treated with atropia sulphate, and were much diminished. On the 30th, the treatment was suspended, and on the 31st, the sweats reappeared.

(Obs.)

I venture to say that the four preceding cases are very favorable to the use of ipecac in the treatment of sweats. In these cases, certainly, the success has been less marked—less decisive, I admit, than in the first six cases; but even the reappearance of the sweats, and their renewed suppression by the same treatment, is still a new argument in favor of the positive action of ipecac.

We see, that in case 8 the sweats quickly disappear, at least in part, but the treatment could not be prolonged, because the patient, who is a hard character, invented I know not what pretexts, to induce us to suspend treatment. Certain it is, that though this patient was treated with all ordinary means, and with no resultant amelioration, yet it is a fact that ipecac did more for him than any other remedy.

In case 7, only one injection was given, and we are justified in believing that the effects would have been most marked if the treatment had been continued. I would say the same with regard to case 10. Case 11 seems to furnish us with an instance in which the ipecac produced no results at all satisfactory; but it may be desirable to recur to the details and see readily at a glance, that if the sweats have not been completely suppressed in this case, it is at least remarkable that they are considerably diminished, and have wholly ceased to inconvenience the patient. Probably the same conditions exist in sweatings as in diarrhœa—conditions necessary to obtain the good effects of this drug, upon which, it perhaps may not be without interest to dwell, later on.

### Group III.

*Cases not successful.*

Case 11. Ward St. Julie; female, bed 2. Admitted Dec. 11, 1873. Pulmonary phthisis, third stage, with lesions far advanced. The disease dates back about four months.

For about three months this patient has, nightly, extremely abundant night sweats, which fatigue and enfeeble her much, compelling her to change her linen many times during the night. Let me say further, that this patient had already been under the care of M. Empis five days, and that she was not, upon coming into this ward, a wholly new patient.

Dec. 11. Ordered a clyster of ipecac at night.

Dec. 12. Patient did not sweat last night; injection was kept from 8 P. M. till 1 A. M.

Dec. 13. No sweats; continue treatment.

Dec. 14. Sweats partially reappeared; double dose of the ipecac in the lavement to-night.

Dec. 16. Less sweating.

Dec. 17. Injections inducing rectal tenesmus; compelled to withhold them.

Till Dec. 20th the sweats were less abundant, and they never returned such as they were upon her coming to the hospital.

Dec. 21. Dyspnœa increased; the patient died from asphyxia, Dec. 23rd.

Autopsy revealed vast cavities in the lungs, but not a single alteration in the alimentary mucous membrane.

Case 12. The patient, the subject of this observation, has already been cited as case No. 2. Sweats copious and cold in the morning, and dating back six months. Does not definitely recollect whether the sweats were suppressed when she took the lavements of ipecac for diarrhœa, but she thinks they were (suppressed).

Nov. 2. Two injections of ipecac ordered.

Nov. 3. No change in the sweats; one clyster at 8 P. M.

Nov. 4. She was unable to hold the injection—let it go.

Nov. 8. Still the same condition of sweating; ordered another lavement, given with Sydenham's laudanum, 10 drops.

Nov. 9. Took a lavement last evening, but her condition became very grave last night, by an attack of suffocation. Died Nov. 13th. Autopsy could not be made.

(Obs.)

I have designated these two cases under the class unsuccessful; still, they ought not to be regarded as absolutely contra-indicating treatment by ipecac. In the first case we have obtained somewhat of a success, since, if the sweats were not wholly suppressed, there was certainly an appreciable amelioration. In the second case, every thing comes within the range of *absolute*, *complete* failure, from the first injection; furthermore, the second one could not be held; and further still, when the third was given, a very grave complication, the result of the progress of the disease itself, for which the ipecac could not to the least degree be held responsible, was developed, and prevented our judging the effect.

It should be further remarked that, in these two cases, we have had to deal with patients in the last stage of the tuberculous cachexia, and when, consequently, absorption of medicines is accomplished with much difficulty.

If we now cast a glance at the twelve cases as a whole, which have preceded, we see, that in a pretty constant manner, ipecac has acted advantageously in the cases where night sweats had become a veritable complication in pulmonary phthisis.

The sweats which we encounter in consumptives are, certainly, often, little abundant, variable from day to day, and not of a marked inconvenience to the patients. I should be able to multiply my observations much, if I had wished to turn my attention to this class of cases, but I have wished to take only grave cases, with copious sweats, and dating, without remission, back to a time long passed. One will be convinced, if he wishes to review my twelve cases, that in this respect it is difficult to pick out the case the most conclusive.

Ipecac was used alone in many cases (Nos. 2, 3, 5, 6, 7, 9, 11, 12). In others (1, 4, 8, 10,) its use had been preceded or followed by other means; and even then, it seems that this may have given the best results.

I have not in my possession the books necessary to make a comparative study between ipecac and other medicines; I am far from wishing to make a trial of any of these; I have often had occasion to see the good effects of tannin and tannate of quinine, and latterly, of atropia sulphate.

My only pretention is, to contest that in rebellious cases, ipecac suppresses the sweats, and that, too, for a time somewhat long, without inconveniencing the patient. It is true that in the study of the therapeutics of the phthisical sweats, as well as in that of any particular disease, there are points which do not seem to sufficiently demand the attention of the experimenter.

Why should this remedy, which succeeds well in one case, fail in an other, when the symptom is designated under the same pathological designation? It is not only an affair of individual predisposition; it is more; it is the varieties of the symptoms themselves, which we do not always possess sufficiently complete.

Let us take, for example, the symptom of sweating in pulmonary phthisis: Sometimes the sweats are partial, sometimes they are general; in some cases they come on in the morning, in others they persist through the night. These are probably not all the varieties, of little importance, but, a fact which seems to me to have been too long neglected, is the following: Are the sweats preceded, or not, by fever? Do they come on after a period of heat, or is it rather the cold sweats which exhaust the patient upon arising from a relatively calm sleep? It is probable that in the two cases the treatment ought not to be the same, and that the same means which succeeds perfectly in the first case, would, in the second, be followed by absolute failure. This is a problem which I leave here, and whose solution, so desirable, would probably give the key to most of the failures. If I

essay within such narrow limits to make an application of these ideas to ipecac, I state, without being able to prove it:

1st. That in most of the cases where ipecac has had a good effect, the fever is noticed preceding the sweats.

2nd. That in cases of failures, and conspicuously in case 12, the fever is wanting.

MODE OF ADMINISTRATION.—DOSES.

In the preceding portion of this article we confined our attention to clinical observations of patients. Now, then, we should turn our attention to the study of the mode of administration, and doses, which is the necessary complement of the clinic.

We have seen that in nearly every case the ipecac was given per rectum. Does that mean, that, administered thus, we hope in diarrhœa to get a local action? We are to infer nothing of the sort. The lesions which produce diarrhœa in the tuberculous, when there are ulcerations, the catarrh, which is encountered only in other cases (this is the sole alteration which we have verified in our autopsies), are located preferably in the small intestine, at a point where liquids, injected into the anus cannot reach. We concluded that the action of ipecac is produced, after absorption, by a special action upon the secretions of the intestinal mucous membrane.

The absorption and acting at a distance have been, we believe, clearly proven in Groups I and II, with regard to the action of sweatings. On the other hand, we wished to avoid vomiting, which was produced almost invariably in the cases where the decoction is administered by the mouth, or in the form of the Brazilian potion. In two cases (Group II, Case IX), where we used this last method, we have seen the vomiting effectively take place. We believe that the administration of ipecac per rectum is a veritable progress, and we hasten to announce that this method is due to our excellent senior, M. Bourdon.

The action being the same where ipecac is given in injection, and the gastric troubles not then existing, it is necessary to know if this medicament, reputed alterative, can be given to patients already enfeebled, in large doses. The affirmative seems to us here to have been given beyond a doubt, by the numerous mentioned cases. The following case of a young infant, feeble and *syphilitic*, who has taken ipecac for a long time, ought to be given to still further confirm this fact:

OBSERVATION:

*Infant of two months; congenitally syphilitic; prolonged diarrhœa; cure.*

Infant of two months; brought up at the breast; pemphigus of the buttocks and thighs; mucous patches at the arms. Admitted to the hospital, with its mother, Oct. 5, 1873.

Oct. 8. Diarrhœa abundant; the infant no longer takes the breast; three injections of ipecac of five grammes (?)

Oct. 9. The same condition; no vomiting; continue.

Oct. 10. General condition better; the same diarrhœa; continue.

Oct. 11. Two injections.

Oct. 29. The general state of the child is good enough; he has diarrhœa daily; he has had two clysters daily, of ten grammes

Oct. 30. Diarrhœa begins to diminish; general condition excellent; the syphilitic concomitants have partially disappeared, and from this time he has resumed anti-syphilitic treatment, suspended since the advent of the diarrhœa.

He left the hospital at the end of November in a good condition.

Here the effect of the ipecac upon the diarrhœa was *nil;* but the point upon which I wish to insist is, the prolongation of treatment without a single sort of accident, either upon the stomach or the intestines, and the perfect general health.

Ipecac can be administered in large doses and for a long time, without producing a single mishap; this is the first point I would establish. Furthermore, its administration by injection is an easy means to employ, and one which possesses, without a single inconvenience, the very same advantages as when presented by the mouth.

The *Decoction of Ipecac* which we have used, in all cases, was prepared in the following manner:

℞ Powdered Ipecac Root, 10 grammes (154 grains).
Pure Water, 100 grammes.
Boil till it is reduced to 30 grammes.
Filter the first decoction.

Prepare a second decoction analogous to the first one; mix the two decoctions and add 5 to 10 drops of Sydenham's laudanum.

The laudanum had only one use, and that was, to guard the injection as long as possible at the same time that it was administered in a small volume. However, the opiates had often failed before the ipecac was used. To guard against any loss of the injection liquid, so little abundant, we had always been careful to use a glass syringe.

The hours chosen were, in diarrhœa, in the morning and evening, at least two hours before or after eating.

The patients who were treated for sweats have, in most instances, taken only a single injection daily, and this was administered as late as possible at night.

In those cases where we used the Brazilian potion, we prepared it as follows:

Decoction of ipecac, prepared as the preceding, but reduced to 45 grammes.
Syrup of ether, 15 grammes.

To be administered in three parts, each a quarter of an hour apart, in the evening.

This is the way in which we have administered ipecac.

One point further, perhaps the most interesting to settle, as to the action of ipecac. By which one of its constituents do we get its therapeutical effects?

We do not yet possess the necessary information to elucidate this question in all its aspects, still here are certainly some results which we have obtained.

The decoction removes from ipecac root many substances, among which are, conspicuously, *tannin* and *emetine.* Is it only by the tannin or the emetine that these decoctions are effective?

In several of our observations on sweatings, tannin in substance and in large doses, (30 to 40 centigrammes $=4\frac{1}{2}$ to 6 grains,) produced no appreciable effects. To admit that the decoction owed its effects to the tannin, it was necessary that the injections should contain a portion of tannin at least equal to that which was administered daily by the mouth.

This it seemed easy to determine by chemical analysis. * * *

Ten grammes of ipecac give the following results:

Solid residue, 3 grammes (45 grains), composed of these ingredients:

Emetine—O. gr. 58 c.=8.7 grains.

Tannin—O. gr. 09 c.=1.3 grains.

Gum,
Starch, } Indefinite.
Cellulose,

A patient treated thus for sweats, absorbs, at most, 1½ grains of tannin, a dose certainly inferior to that which we gave in pill by the mouth.

Must we attribute this advantageous effect then to emetine? Emetine is a substance whose physiological properties are but little known. We certainly see no such results here as experiments with it yield. * * *

The only results I wish to give to-day are the following:

1st. Ipecac, even in large doses, administered per rectum, does not produce vomitings or gastric disturbance.

2d. The action of injections of ipecac upon diarrhœa of young infants has seemed to us, in many instances, very favorable.

3d. Diarrhœa of the tuberculous was beneficially influenced, often cured for a long time, by ipecac in large doses. We must add that, in all cases where, after cure of the diarrhœa, we made autopsies, we found no organic lesions of the mucous membrane.

4th. The action of ipecac upon phthisical sweats has been the most frequently favorable.

5th. Ipecac seems to act by absorption.

6th. The tannin is in too small a quantity in the decoction to have the therapeutical effect obtained attributed to it.

---

Bell on Aspiration in Retention of Urine.—Dr. Joseph Bell relates an instructive case (*Edinburg Medical Journal*, April, 1874), and adds: Cases admitting or requiring this treatment, will not likely be very frequent—indeed I have not met with another out of a very large number of stricture cases seen since June; still, in this one, any other treatment would have been very dangerous.

Perineal section is always tedious, requiring chloroform, which the weak heart and emphysematous lungs and diseased kidneys would have borne ill; besides perineal section has its own dangers in old exhausted subjects. Tapping by rectum would have been difficult, from the enlarged prostate. Catheterization had failed. The operation was painless and left no trace. I have a very strong feeling that, in similar cases, the aspirator gives us an easy, safe and reliable means of tiding over a difficulty, emptying the bladder, and thus giving time for other treatment. It is possible, if necessary, to repeat the aspiration frequently in the same region, but not exactly in the same situation.

The special merit of the aspirator here is, that it enables us, by the suction power it possesses, to withdraw the urine through a tube little larger than an acupressure needle, the wound inflicted by which heals up at once and leaves no trace.

---

The Action of Bromide of Calcium and of Bromide of Potassium.—The former salt acts only on the nerves, but it produces less sedative effect than the potassium bromide, and it does not act at all on the heart, as does the latter salt.—*Guttmann and Eulenburg*—*Ber. Klin. Wochenschrift.*

